# Aerobic Pathogens and Antimicrobial Susceptibility in Odontogenic Infections: A One-Year Observational Study from Southwestern Romania

**DOI:** 10.3390/medicina61112008

**Published:** 2025-11-10

**Authors:** Horatiu Urechescu, Marius Pricop, Victor Vlad Costan, Silvia Oniga, Cristiana Cuzic, Ancuta Banu

**Affiliations:** 1 Department of Oral and Maxillo-Facial Surgery, Faculty of Dental Medicine, University of Medicine and Pharmacy “Victor Babes”, 300041 Timisoara, Romania; urechescu.horatiu@umft.ro (H.U.);; 2 Department of Surgery, Faculty of Dental Medicine, University of Medicine and Pharmacy “Gr. T. Popa”, 700115 Iasi, Romania; 3 Department of Prosthodontics, Faculty of Dental Medicine, University of Medicine and Pharmacy “Victor Babes”, 300041 Timisoara, Romania

**Keywords:** odontogenic infections, aerobic bacteria, antimicrobial susceptibility

## Abstract

*Background and Objectives:* Odontogenic infections are common emergencies in oral and maxillofacial surgery. They are typically polymicrobial, with aerobes guiding initial empirical therapy. However, regional data on their microbiology and resistance patterns in Romania are limited. This study aimed to characterize the aerobic microbial profile of odontogenic infections in Southwestern Romania and assess the antimicrobial susceptibility of isolated pathogens. *Materials and Methods:* A prospective observational study was conducted over 12 months at a tertiary referral hospital. Pus samples collected intraoperatively were cultured aerobically. Bacterial identification used biochemical methods and the VITEK 2 system. Antimicrobial susceptibility was determined by disk diffusion and automated MIC testing, interpreted according to EUCAST v13.0 (2023). *Results:* Of 110 patients, 96 (87.3%) yielded positive aerobic cultures, producing 97 isolates. Streptococcus spp. were predominant (49.5%), followed by coagulase-negative staphylococci (24.7%), *Staphylococcus aureus* (14.4%), *Enterobacterales* (7.2%), and *Pseudomonas aeruginosa* (3.1%). *Streptococcus* spp. remained susceptible to penicillin G (82.3%), amoxicillin–clavulanate (76.4%), and clindamycin (70.5%), but only 55.0% to erythromycin. Most *S. aureus* isolates were methicillin-susceptible (92.9%), while coagulase-negative staphylococci showed high methicillin resistance (59.3%) yet full susceptibility to linezolid, vancomycin, and teicoplanin. *Enterobacterales* were resistant to ampicillin (90%) and amoxicillin–clavulanate (65%) but remained susceptible to ceftriaxone (80%) and ciprofloxacin (85%). *P. aeruginosa* isolates were fully susceptible to piperacillin–tazobactam, ceftazidime, cefepime, and meropenem. *Conclusions:* This study provides regional data on aerobic pathogens in odontogenic infections. High resistance to penicillin and macrolides limits empirical use. Amoxicillin–clavulanate and clindamycin retain moderate activity, while glycopeptides, linezolid, and carbapenems preserved full efficacy. Surgical drainage remains central to management, and antibiotic therapy should be guided by local susceptibility patterns. These data provide baseline information to inform empirical therapy and stewardship efforts and highlight the need for multicenter studies including anaerobic and molecular analyses.

## 1. Introduction

Odontogenic infections are among the most frequent causes of head-and-neck suppurative disease managed in oral and maxillofacial surgery, ranging from localized periapical abscesses to deep fascial space infections with potential airway compromise, descending necrotizing mediastinitis, intracranial complications, and sepsis. Despite advances in dental care and antimicrobial therapy, these infections remain a common indication for urgent admission and surgical drainage in tertiary centers. Their polymicrobial nature and the increasing prevalence of antimicrobial resistance complicate empirical antibiotic selection and underscore the need for up-to-date, region-specific microbiological data to inform stewardship.

Classical culture-based studies established the early role of viridans group streptococci and *Streptococcus milleri* complex in odontogenic infections and documented the later recognition of anaerobes as cultivation methods and transport systems improved [1,2,3,4,5,6]. The introduction of molecular microbial detection techniques further expanded the recognized diversity of odontogenic infections, revealing communities substantially richer than those detected by conventional culture and highlighting transitions from health to disease states within the oral microbiome [7,8,9,10,11]. While historically informative, these data predate contemporary antibiotic exposure patterns and today’s resistance ecology.

Recent clinical and genomic studies confirm that odontogenic infections remain polymicrobial, with aerobic/facultative streptococci and *Staphylococcus aureus* frequently recovered alongside anaerobes such as *Prevotella*, *Fusobacterium*, and *Peptostreptococcus* in advanced disease. Multi-site surveillance in Europe has demonstrated substantial regional variation in resistance among oral pathogens and rising rates of β-lactam and macrolide resistance [12,13,14,15,16]. Next-generation sequencing analyses further reveal mixed microbial communities and novel resistance determinants not detectable by standard culture [15]. These data reinforce the need for updated, locally grounded microbiological baselines to support rational empiric antibiotic therapy.

At the population level, antibiotic consumption in Europe rebounded after the initial pandemic decline, and inappropriate use continues to drive antimicrobial resistance. ESAC-Net and EARS-Net reports (ECDC, 2023) show persistently high consumption of broad-spectrum agents, especially in southern and eastern Europe, with Romania remaining among the highest for penicillins and fluoroquinolones [17,18,19]. These trends directly influence local susceptibility profiles observed in dental and maxillofacial infections.

In Romania, recent culture-based data on aerobic pathogens in odontogenic infections are lacking. The Southwestern region reports particularly high community antibiotic use and resistance levels [19]. Tertiary oral and maxillofacial units in this area manage numerous severe odontogenic infections, yet no standardized, prospective microbiological analysis has been published in the post-COVID era. A current, region-specific dataset is therefore essential to guide empiric therapy and antibiotic stewardship in this setting.

Although obligate anaerobes are important contributors to advanced odontogenic infections, aerobic and facultative organisms are typically recovered in early stages and drive initial empiric management [14,17]. Routine anaerobic culture was limited in our hospital during the study period due to resource constraints, and anaerobic AST generally requires prolonged turnaround times. Thus, we focused on aerobic bacteria using standardized EUCAST-based testing to generate data immediately relevant for empirical decision-making, while acknowledging that comprehensive studies including anaerobes and molecular assays remain necessary.

This study aimed to characterize the aerobic microbial profile and antimicrobial susceptibility patterns of odontogenic infections requiring surgical management at a tertiary referral center in Southwestern Romania over a 12-month period. The results provide current, region-specific baseline data to inform empirical antibiotic selection and support stewardship programs.

## 2. Materials and Methods

### 2.1. Study Design and Setting

We conducted a prospective observational study over a 12-month period (January–December 2024) at the Department of Oral and Maxillofacial Surgery, Municipal Emergency Hospital, Timisoara (Romania). The study protocol was reviewed and approved by the Institutional Ethics Committee (E-3789/2025). All procedures were conducted in accordance with the Declaration of Helsinki. Written informed consent was obtained from all patients (or their guardians, where applicable).

### 2.2. Patient Selection

The study included patients admitted with acute odontogenic infections requiring surgical intervention. Inclusion criteria were clinical diagnosis of odontogenic infection, indication for surgical drainage or tooth extraction and absence of severe systemic comorbidities that might independently compromise immunity. Exclusion criteria included: infections of non-odontogenic origin, prior hospitalization for the same infection and inability to obtain appropriate biological samples. Demographic and clinical data (age, sex, site of infection, prior antibiotic use, length of hospital stay) were collected from medical records. Detailed information on prior outpatient antibiotic exposure was not consistently available and therefore was not analyzed statistically. The final sample size (*n* = 96) included all consecutive culture-positive odontogenic infection cases admitted during the 12-month period; therefore, no additional statistical sampling was applied.

### 2.3. Sample Collection and Transport

Pus specimens were collected intraoperatively, preferentially by needle aspiration into sterile syringes. When aspiration was not feasible, sterile cotton swabs were used. Approximately two-thirds of the 96 culture-positive specimens were aspirates and one-third were swabs, reflecting technical feasibility during drainage procedures. To minimize contamination with oral flora, sampling was performed only after surgical access was established. All specimens were placed in sterile transport medium and delivered to the microbiology laboratory within 2 h of collection and were maintained at room temperature during transport. Differences in collection technique may influence bacterial yield, and this heterogeneity was considered in the interpretation of organism distribution.

### 2.4. Microbiological Processing and Identification

Samples were inoculated onto blood agar, chocolate agar, and MacConkey agar plates and incubated aerobically at 35–37 °C for 24–48 h. This study focused on aerobic and facultative organisms because these are typically the first pathogens recovered in early-stage odontogenic infections and therefore have direct empirical relevance for initial antibiotic therapy. Routine anaerobic culture and susceptibility testing were not consistently feasible during the study period due to limited access to specialized anaerobic transport and incubation systems. As a result, the microbiological profile presented here reflects the aerobic component of odontogenic infections and should be interpreted within this scope. Colonies were identified by standard biochemical methods and confirmed using the VITEK 2 Compact system (bioMérieux, Craponne, France; software version 9.02).

Identification results from the VITEK 2 Compact system were verified by parallel conventional biochemical tests in cases of ambiguous profiles, and internal performance checks were conducted daily in accordance with manufacturer and EUCAST recommendations.

### 2.5. Antimicrobial Susceptibility Testing (AST)

Antimicrobial susceptibility was determined by the Kirby–Bauer disk diffusion method on Mueller–Hinton agar and, where required, by automated minimum inhibitory concentration (MIC) determination with the VITEK 2 Compact. Bacterial suspensions were standardized to a 0.5 McFarland density. Plates were incubated at 35 °C for 18–24 h. Zone diameters and MICs were interpreted according to the European Committee on Antimicrobial Susceptibility Testing (EUCAST) clinical breakpoints, version 13.0 (2023). Results were interpreted according to EUCAST 2023 criteria, where categories were defined as susceptible (S), susceptible at increased exposure (I), or resistant (R). Not all isolates underwent full susceptibility testing due to duplicate strains, mixed cultures, or insufficient growth. The number of isolates tested for each antibiotic is indicated in the corresponding tables.

### 2.6. Quality Control

Quality-control procedures were performed daily, using the following reference strains recommended by EUCAST: *Staphylococcus aureus* ATCC 25923 and *Escherichia coli* ATCC 25922 for disk diffusion; *S. aureus* ATCC 29213 and *E. coli* ATCC 35218 for MIC determination.

### 2.7. Data Analysis

All data were entered into a secure database and analyzed using IBM SPSS Statistics (version 26, IBM Corp., Armonk, NY, USA). Continuous variables were reported as mean ± standard deviation (SD). Categorical variables were summarized as absolute counts and percentages. Differences in organism distribution and resistance rates across clinical subgroups were assessed using the Chi-square test. Proportions were presented with 95% confidence intervals (CI). A *p*-value < 0.05 was considered statistically significant. Multivariate analysis was not performed due to the exploratory nature of the study and the limited sample size, which did not allow reliable adjustment for multiple covariates. Further inferential testing was not feasible due to small subgroup counts, and multivariate analysis was not performed because of the limited sample size and exploratory design of the study.

## 3. Results

Over the one-year study period, 110 patients with acute odontogenic infections were admitted to the Oral and Maxillofacial Surgery Department. Of these, 96 (87.27%) yielded positive aerobic cultures and were included in the microbiological analysis.

Fourteen samples (12.7%) showed no growth, likely due to prior antibiotic exposure or sampling/transport limitations.

The mean age of patients with positive cultures was 40.2 ± 17.2 years (range 18–77 years), with a male-to-female ratio of 1.4:1. Of these, 38 patients (33.9%) were under 30 years of age, 11 (9.8%) were aged ≥ 65, and the majority (47; 49.0%) were aged 31–65 years.

The most frequent anatomical site of infection was the peri-maxillary bone (*n* = 46, 41.1%), followed by primary fascial spaces (n = 36, 32.1%), secondary fascial spaces (*n* = 9, 8.4%), and phlegmons (*n* = 5, 4.5%) (Table 1).

No statistically significant differences in localization were observed based on sex (χ^2^ = 2.00, *p* = 0.158) and the age distribution did not differ significantly among infection types (χ^2^ = 1.28, *p* = 0.53).

A total of 97 aerobic bacterial isolates were recovered from the 96 culture-positive patients.

The distribution of organisms by infection site is summarized in Table 2. The predominant organisms were *Streptococcus* spp. (48 isolates; 49.5%), followed by coagulase-negative staphylococci (24; 24.7%), *Staphylococcus aureus* (14; 14.4%), *Enterobacterales* (7; 7.2%), and *Pseudomonas aeruginosa* (3; 3.1%). Streptococci predominated in peri-maxillary and primary fascial infections, whereas *S. aureus* and Gram-negative bacilli were more frequent in secondary-space and phlegmonous infections (χ^2^ = 7.84, *p* = 0.02). An exploratory Chi-square analysis comparing bacterial groups across age categories showed no significant association (χ^2^ = 1.32, *p* = 0.25). No significant correlation was observed between infection site and antimicrobial resistance pattern (*p* > 0.05), likely reflecting the limited subgroup sizes.

Susceptibility testing followed EUCAST v13.0 standards. Results for Gram-positive and Gram-negative isolates are shown in Table 3 and Table 4, with 95% confidence intervals (CIs) for key agents. Of the 48 Streptococcus isolates identified, 34 yielded complete susceptibility data suitable for analysis; missing cases represented duplicate strains or insufficient growth.

### 3.1. Gram-Positive Isolates

*Streptococcus* spp. remained highly susceptible to penicillin G (82.3%; 95% CI 68.0–91.4) and amoxicillin–clavulanate (76.4%; 95% CI 61.2–87.4), with moderate susceptibility to clindamycin (70.5%; 95% CI 54.4–83.1) and reduced susceptibility to erythromycin (55.0%; 95% CI 39.8–69.3).

Among *S. aureus* isolates, 92.9% were methicillin-susceptible (one MRSA isolate). All *S. aureus* and coagulase-negative staphylococci were fully susceptible to linezolid, vancomycin, and teicoplanin. CoNS exhibited 59.3% methicillin resistance but complete susceptibility to glycopeptides and tigecycline.

### 3.2. Gram-Negative Isolates

Resistance among Gram-negative organisms was confined mainly to β-lactams. *Enterobacterales* were resistant to ampicillin (90%) and amoxicillin–clavulanate (65%), but remained susceptible to ceftriaxone (80%) and ciprofloxacin (85%). *P. aeruginosa* isolates were fully susceptible to piperacillin–tazobactam, ceftazidime, cefepime, and meropenem, with one isolate showing reduced susceptibility to ciprofloxacin. Clindamycin testing was not performed for Gram-negative isolates, as the drug has no activity against them.

No statistically significant association was found between the type of bacterial isolate (Gram-positive vs. Gram-negative) and the patient’s age group or infection site (*p* > 0.05).

*Streptococcus* spp. were the predominant aerobic pathogens, representing roughly half of all isolates. Penicillin and macrolides showed reduced efficacy, whereas amoxicillin–clavulanate and clindamycin retained moderate activity against Gram-positive organisms. Reserve agents (linezolid, vancomycin, tigecycline) remained universally active. Gram-negative isolates, although less frequent, demonstrated marked resistance to narrow-spectrum β-lactams but preserved susceptibility to third-generation cephalosporins, fluoroquinolones, and carbapenems. Secondary and phlegmonous infections were more likely to yield Gram-negative or resistant organisms (*p* < 0.05). Exploratory analyses found no significant correlation between patient age or infection site and resistance pattern (*p* > 0.05).

These findings support amoxicillin–clavulanate as a reasonable empirical therapy for non-allergic patients and clindamycin as an appropriate alternative in β-lactam–allergic individuals, with local resistance rates (~30%) warranting close clinical monitoring.

## 4. Discussion

This study characterized the aerobic microbial profile and antimicrobial susceptibility of odontogenic infections managed surgically in a tertiary center in Southwestern Romania. We found that streptococci predominated in peri-maxillary and primary fascial space infections, while staphylococci and Gram-negative species were more frequent in secondary spaces and phlegmons. These findings are consistent with previous observations that streptococci initiate the infectious process and facilitate anaerobic overgrowth in later stages [5,20,21,22]. Similar to other published data [23], the localization of suppurative processes did not depend on gender but varied with age, with greater severity in older patients.

Exploratory Chi-square analyses did not identify significant associations between age group and bacterial type (χ^2^ = 1.32, *p* = 0.25) or between infection site and resistance pattern (*p* > 0.05), suggesting that bacterial distribution and resistance in this cohort were not strongly influenced by demographic variables.

It is important to note that our results reflect the aerobic spectrum only. Anaerobes are well recognized as major contributors to odontogenic infections, particularly in advanced fascial space disease, but their isolation requires specialized facilities not consistently available in our setting. Consequently, our analysis focused on aerobes, which provide the most immediate guidance for empiric therapy. This methodological limitation may reduce the completeness of our microbiological profile and should temper interpretation of our results, as obligate anaerobes such as *Prevotella*, *Fusobacterium*, and *Peptostreptococcus* often predominate in advanced disease [5,6,20,21,22]. Nevertheless, aerobic bacteria are usually the first pathogens isolated in early stages and remain highly relevant for guiding empiric therapy, especially before full culture results are available.

The susceptibility results highlight concerning resistance trends. *Streptococcus* spp. retained activity to penicillin G (82.3%), amoxicillin–clavulanate (76.4%), and clindamycin (70.5%), but resistance to erythromycin was substantial. Comparable resistance patterns have been reported in other European cohorts, with similar macrolide and clindamycin resistance levels among oral and maxillofacial isolates [14,15]. *Staphylococcus aureus* isolates were mostly methicillin-susceptible, with a single MRSA isolate detected, while coagulase-negative staphylococci showed high rates of methicillin resistance. All Gram-positive isolates remained susceptible to linezolid, vancomycin, and teicoplanin. Gram-negative bacilli demonstrated high resistance to ampicillin (90%) and amoxicillin-clavulanate (65%), but lower resistance to ceftriaxone (20%) and ciprofloxacin (15%). *Pseudomonas aeruginosa* isolates were uniformly susceptible to piperacillin-tazobactam, ceftazidime, cefepime, and meropenem, with one ciprofloxacin-resistant strain.

These resistance patterns are consistent with national antimicrobial consumption trends in Romania, where penicillins—particularly amoxicillin–clavulanate—remain among the most frequently prescribed agents [13,24]. The high rate of resistance to amoxicillin–clavulanate observed in our study reflects this extensive use. Similarly, elevated erythromycin resistance corresponds to historical macrolide overuse, whereas the preserved activity of third-generation cephalosporins and aminoglycosides parallels their lower national consumption and recent declines in use [13]. Carbapenem prescriptions have increased substantially in recent years, yet no carbapenem resistance was identified in our isolates. Quinolone resistance remained moderate despite sustained use and recent regulatory restrictions [13]. While these parallels between national antibiotic consumption and local resistance patterns are informative, they should be regarded as contextual rather than causal, given that our dataset derives from a single regional population.

The presence of methicillin-resistant coagulase-negative staphylococci (24.1%) is clinically relevant, as these strains often exhibit cross-resistance to multiple classes. In our series, 76.5% were resistant to erythromycin and 35.3% to clindamycin, but all remained susceptible to vancomycin. Fusidic acid activity was noted, although its toxicity limits clinical applicability. Cotrimoxazole demonstrated good in vitro activity, which may relate to its reduced clinical use in Romania. These findings emphasize the importance of preserving reserve agents for severe or resistant infections.

From a clinical standpoint, our results suggest that penicillin and macrolides are no longer reliable for empirical coverage in our region. Clindamycin and amoxicillin-clavulanate may be more effective alternatives but should be used cautiously given emerging resistance. Surgical drainage remains the cornerstone of therapy, while antibiotics serve as adjunctive treatment guided by culture and local epidemiology.

Several limitations should be acknowledged. This was a single-center study with a modest sample size, which limited analytical depth and precluded multivariate analysis. The exclusion of anaerobic cultures restricts the scope of our findings. Some patients had received antibiotics before admission, which may have influenced culture yield, and detailed data on prior exposure were incomplete. Molecular testing to confirm resistance mechanisms was not performed. Our analysis did not correlate microbiological findings with patient outcomes or treatment responses, which limits the assessment of clinical impact and should be addressed in future prospective studies. Although the study covered a full 12-month period, no temporal or seasonal analysis of infection patterns or resistance was conducted. While aspiration was the preferred method of pus collection, sterile swabs were used in cases where aspiration was technically difficult or unsafe. Approximately two-thirds of the culture-positive specimens were aspirates and one-third were swabs. Swab sampling has a lower yield and higher risk of contamination from oral flora compared to aspirates [5,6], but all samples were collected intraoperatively under sterile conditions and processed promptly, minimizing this bias. Cultures were qualitative rather than quantitative, limiting assessment of bacterial dominance. Statistical analysis was limited to descriptive and Chi-square methods; exploratory tests did not reveal significant associations between patient variables and microbial distribution. These limitations highlight the need for future multicenter research integrating both aerobic and anaerobic isolates, quantitative and molecular methods, and clinical correlations to refine empiric therapy in odontogenic infections.

Comparative surveys on antibiotic prescribing in oral surgery have reported frequent empirical use of broad-spectrum agents and variable adherence to prophylactic guidelines [25,26,27,28,29]. In line with these observations, our results underscore the ongoing need for rational antibiotic selection and adherence to stewardship principles in dental and oral-surgical infections. Continuous evaluation of prescribing practices and the implementation of region-specific empirical protocols based on local resistance data are essential to support evidence-based management of odontogenic infections.

## 5. Conclusions

This one-year observational study provides updated regional data on the aerobic microbiology and antimicrobial susceptibility of odontogenic infections in Southwestern Romania. Streptococcus species predominated in peri-maxillary and primary fascial space infections, whereas Staphylococcus aureus and Gram-negative bacilli were more frequently isolated from secondary-space and phlegmonous infections. High resistance rates to penicillin and macrolides were observed, while amoxicillin–clavulanate and clindamycin retained moderate activity. All isolates were fully susceptible to glycopeptides and linezolid.

Surgical drainage remains the cornerstone of treatment for odontogenic infections, with antibiotic therapy serving as an essential adjunct guided by local susceptibility patterns and infection site. The present findings provide baseline data that may assist clinicians in selecting empiric antibiotics while culture results are pending. However, the results should be interpreted within the context of the study’s limitations, including its single-center design, modest sample size, and the absence of anaerobic culture data.

Future multicenter studies incorporating both aerobic and anaerobic isolates, quantitative or molecular methods, and larger sample sets are warranted to strengthen epidemiologic understanding and guide evidence-based antimicrobial stewardship in oral and maxillofacial infections.

## Figures and Tables

**Table 1:** Demographic characteristics and infection site distribution among 96 patients with aerobic culture–positive odontogenic infections.

Infection Type	≤30 y (*n*)	31–65 y (*n*)	≥65 y (*n*)	Total (*n*)	% of Total
Peri-maxillary	21	21	4	46	47.9%
Primary fascial spaces	12	21	3	36	37.5%
Secondary fascial spaces	3	3	3	9	9.4%
Phlegmons	2	2	1	5	5.2%
Total	38	47	11	96	100%

**Table 2:** Distribution of aerobic bacterial isolates (*n* = 97) by infection site.

Infection Type	*Streptococcus* spp.	*S. aureus*	CoNS	Enterobacterales	*P. aeruginosa*	Others	Total
Peri-maxillary	24	6	10	4	1	1	46
Primary fascial spaces	21	5	8	1	1	0	36
Secondary fascial spaces	2	2	4	1	0	0	9
Phlegmons	1	1	2	1	1	0	6
Total	48	14	24	7	3	1	97

*Abbreviations:* CoNS = coagulase-negative staphylococci.

**Table 3:** Antimicrobial susceptibility of Gram-positive aerobic isolates from odontogenic infections.

Gram-Positive Isolates	Antibiotic	*n* Tested	% Susceptible (95%CI)
*Streptococcus* spp.	Penicillin G	34	82.3% (68.0–91.4)
	Amoxicillin-clavulanate	34	76.4% (61.2–87.4)
	Clindamycin	34	70.5% (54.4–83.1)
	Erythromycin	34	55.0% (39.8–69.3)
Staphylococcus aureus	Oxacillin	14	92.9% (66.1–99.8)
	Clindamycin	14	78.6% (49.2–95.3)
	Linezolid, Vancomycin, Teicoplanin	14	100%
CoNS	Oxacillin	27	40.7% (22.4–61.2)
	Clindamycin	27	66.7% (45.4–84.3)
	Linezolid, Vancomycin, Teicoplanin, Tigecycline	27	100%

*Note:* Of 48 Streptococcus isolates identified, 34 produced complete and valid susceptibility results; minor discrepancies reflect duplicate or technically invalid tests.

**Table 4:** Antimicrobial susceptibility of Gram-negative aerobic isolates from odontogenic infections.

Gram-Negative Isolates	Antibiotic	*n* Tested	% Susceptible (95% CI)
Enterobacterales	Ampicillin	7	10% (0.3–44.5)
	Amoxicillin-clavulanate	7	35% (7.5–70.1)
	Ceftriaxone	7	80% (44.4–97.5)
	Ciprofloxacin	7	85% (47.9–99.2)
Pseudomonas aeruginosa	Piperacillin-tazobactam	3	100%
	Ceftazidime	3	100%
	Cefepime	3	100%
	Meropenem	3	100%
	Ciprofloxacin	3	100%

*Note:* Number of isolates tested varies due to occasional growth failure or mixed cultures. Results for Pseudomonas aeruginosa are based on three isolates. Percentage values should be interpreted with caution due to the small sample size.

## Data Availability

The data generated in this study may be requested from the corresponding author.
